# Vitamin C supplement use may protect against gallstones: an observational study on a randomly selected population

**DOI:** 10.1186/1471-230X-9-74

**Published:** 2009-10-08

**Authors:** Thomas Walcher, Mark M Haenle, Martina Kron, Birgit Hay, Richard A Mason, Daniel Walcher, Gerald Steinbach, Peter Kern, Isolde Piechotowski, Guido Adler, Bernhard O Boehm, Wolfgang Koenig, Wolfgang Kratzer

**Affiliations:** 1 Department of Internal Medicine II, University Hospital Ulm, Ulm, Germany; 2 Department of Internal Medicine I, University Hospital Ulm, Ulm, Germany; 3 Department of Biometry and Medical Documentation, Ulm University, Ulm, Germany; 4 Department of Veterans Affairs, Louis Stokes Cleveland Medical Center, Brecksville Division, Brecksville, Ohio 44141, USA; 5 Department of Clinical Chemistry and Pathobiochemistry, University Hospital Ulm, Ulm, Germany; 6 Department of Internal Medicine III, Division of Infectious Diseases and Clinical Immunology, University Hospital Ulm, Ulm, Germany; 7 Baden-Württemberg State Health Office, District Government Stuttgart, Stuttgart, Germany

## Abstract

**Background:**

Animal experiments have shown a protective effect of vitamin C on the formation of gallstones. Few data in humans suggest an association between reduced vitamin C intake and increased prevalence of gallstone disease. The aim of this study was to assess the possible association of regular vitamin C supplementation with gallstone prevalence.

**Methods:**

An observational, population-based study of 2129 subjects aged 18-65 years randomly selected from the general population in southern Germany was conducted. Abdominal ultrasound examination, completion of a standardized questionnaire, compilation of anthropometric data and blood tests were used. Data were collected in November and December 2002. Data analysis was conducted between December 2005 and January 2006.

**Results:**

Prevalence of gallstones in the study population was 7.8% (167/2129). Subjects reporting vitamin C supplementation showed a prevalence of 4.7% (11/232), whereas in subjects not reporting regular vitamin C supplementation, the prevalence was 8.2% (156/1897). Female gender, hereditary predisposition, increasing age and body-mass index (BMI) were associated with increased prevalence of gallstones. Logistic regression with backward elimination adjusted for these factors showed reduced gallstone prevalence for vitamin C supplementation (odds ratio, OR 0.34; 95% confidence interval, CI 0.14 to 0.81; P = 0.01), increased physical activity (OR 0.62; 95% CI, 0.42 to 0.94; P = 0.02), and higher total cholesterol (OR 0.65; 95% CI, 0.52 to 0.79; P < 0.001).

**Conclusion:**

Regular vitamin C supplementation and, to a lesser extent, increased physical activity and total cholesterol levels are associated with a reduced prevalence of gallstones. Regular vitamin C supplementation might exert a protective effect on the development of gallstones.

## Background

Disorders of the gallbladder are a major cause of morbidity and a leading indication for hospital admissions in both the United States and Europe. The economic impact of gallstone disease in Western industrialized countries is high [1,2].

Clinical and experimental data reported in the 1970's suggested a potential protective effect of vitamin C on the formation of gallstones [3,4]. Furthermore, animal experiments have shown that animals deficient in vitamin C more frequently develop gallstones [5,6].

The role of vitamin C in bile acid biogenesis has mainly been analysed in guinea pigs [3-9]. Cholesterol is converted to bile acids in the liver, and the rate limiting process depends on vitamin C concentration in the hepatocytes [3,4]. Vitamin C increases the rate of 7α-hydroxylation of cholesterol [4,7,8]. This reaction is decreased in ascorbic acid deficiency, resulting in reduced bile acid biogenesis [4,9]. Supersaturation of bile with cholesterol precedes the formation of cholesterol gallstones and this can be caused by a low rate of cholesterol-7α-hydroxylation [10]. Guinea pigs receiving ascorbic acid substitution showed a 15-fold increase in the activity of cholesterol-7α-hydroxylase compared to those deficient in ascorbic acid [7]. Ascorbic acid deficient animals more frequently developed cholesterol gallstones [5,6].

A similiar biochemical explanation for increased development of gallstones in subjects with vitamin C deficiency as described in guinea pigs might exist in humans [11]. While Duane et al. showed that short-term subclinical vitamin C deficiency in five healthy volunteers did not increase the lithogenic potential of gallbladder bile as it did in guinea pigs fed a high cholesterol diet, Gustafson et al. described changes in the bile salt composition and biliary phospholipid levels of vitamin C treated cholesterol gallstone patients and also found support for the notion that vitamin C supplementation might influence the conditions of cholesterol gallstone formation in humans [12,13]. In humans, observational studies have also suggested an association between vitamin C intake and gallstone disease [14-17].

The prevalence of gallstone disease in relation to vitamin C intake has not been studied using ultrasonography in a randomly selected population. The objective of the present study was to evaluate the potential association of regular vitamin C supplement use on gallstone prevalence, as assessed by ultrasonography and patient's history, in a cross-sectional survey of randomly selected subjects from the general population. In addition, we sought to evaluate the effect of other potential risk factors for gallstone disease.

## Methods

### Study population/Subjects

A total of 4,000 subjects aged 10 to 65 years were randomly selected from a target population of 12,475 inhabitants of the city of Leutkirch, Germany for participation in a health survey in November and December 2002 [18]. The survey included among other objectives the possible association of vitamin C as well as the association of the Arg64 variant of the beta3-adrenergic receptor [19] on gallstone prevalence. Out of the 4,000 subjects, 107 were excluded for various reasons (unknown current address, n = 68; not giving informed consent, n = 39). Of 3,893 eligible subjects, 2,445 finally participated in the study (response rate: 62.8%).

Subjects <18 years (n = 258) were excluded in order to enhance comparability with other studies. In addition, subjects were excluded for the following reasons: inability to adequately assess the gallbladder or otherwise limited ultrasound examination conditions (n = 26), highly contracted gallbladder due to inadequate pre-examination fasting period (n = 9), cholecystectomy for gallbladder polyps (n = 4), or refusal to undergo ultrasound examination (n = 1). Subjects unable to provide information on vitamin C supplementation (n = 18) were also excluded. Thus, the final study population consisted of 2,129 subjects (1,025 males, 48.1%; 1,104 females, 51.9%). The study was conducted in accordance with the principles of the Helsinki Declaration and Good Clinical Practice. It was approved by the ethics committee of the Landesärztekammer Baden-Württemberg. All subjects provided written informed consent.

### Survey methods

All subjects completed a standardized questionnaire under the supervision of a trained interviewer, including demographic data, data on medical history, physical activity, dietary habits (use of supplements, duration and amount of alcohol consumption), smoking habits, family history (gallstones, diabetes mellitus, malignancies), and drug history. Further questions on regular vitamin C supplementation (yes/no and daily/weekly intake) and the duration of supplementation (<1 year, 1-5 years, >5-10 years, >10 years) were included. Moreover, subjects were asked about regular medication intake including all vitamin supplementation products (drug name, dosage during the last two weeks).

Body height and weight as well as waist and hip circumference were determined, and body-mass index (BMI) and waist-to-hip ratio (WHR) were calculated [20].

Data analysis was conducted between December 2005 and January 2006.

### Laboratory measurement

Venous blood was drawn with minimal suction under standardized conditions after at least a 4 hour fast. Total cholesterol, high-density lipoprotein (HDL) cholesterol and triglycerides were determined enzymatically on a Dimension XL (Dade Behring Inc., Newark, DE, U.S.A). Low-density lipoprotein (LDL) cholesterol was calculated by the Friedewald formula [21].

### Ultrasonography

Ultrasonography was performed by specially trained clinical investigators. In case of disagreement between the first and the second observer an experienced supervisor (> 4000 ultrasound examinations per year) made the final decision. Examinations were performed using four identical HDI 5000 ultrasound scanners (2-5 MHz probe) provided by Advanced Technology Laboratories Ultrasound (Philips Medical Systems, P.O. Box 3003, Bothell, WA, USA). All subjects underwent ultrasound examination under standardized conditions. If gallstones were detected, the ability to mobilize the gallstones was tested. If differentiation between immobile gallstones and adherent polyps was difficult, patients were re-examined in standing position. Diagnosis of gallstones was made on the basis of at least one of the following criteria: One or more hyperechoic structures in the gallbladder with dorsal shadow; one or more hyperechoic shadows in the gallbladder without dorsal shadow, which by examination in multiple planes or by attempted mobilization could be clearly differentiated from other structures; one strongly hyperechoic structure in the area of the gallbladder with dorsal shadow but in which the gallbladder lumen is no longer or only barely visualized; one or more hyperechoic structures with or without dorsal shadow in the biliary system; no visualization of the gallbladder lumen in patients following cholecystectomy for gallstone disease; presence of a large amount of gallbladder sludge that fills at least one-quarter of the gallbladder lumen with corresponding dorsal shadow.

### Statistical analysis

Absolute and relative frequencies were calculated for categorical variables. For continuous variables, mean and standard deviation, and the median, as well as 5^th ^and 95^th ^percentile were calculated.

Multiple logistic regression was performed in order to identify risk factors associated with prevalence of gallstones [22]. First, the effect of the established risk factors age, gender, BMI and family history were evaluated. Then, for each of the following factors vitamin C supplementation, physical activity, smoking, caffeine and alcohol consumption, total cholesterol, HDL-cholesterol and LDL-cholesterol concentrations, vegetarian diet, diabetes mellitus, waist-to-hip ratio, and inflammatory bowel disease a multiple logistic model adjusted for age, gender, BMI, and family history was fitted to identify further potential risk factors. All factors with P-values < 0.1 were pre-selected. The pre-selection level of 10% was used in order to ensure that important factors will not be overlooked. Pre-selected factors were included in a multiple logistic regression using backward elimination (selection level 5%) in order to identify important risk factors. During the modeling process, age, gender, BMI, and family history were forced into the model. Results are given as Odds Ratios (OR) together with their 95% confidence intervals (CI) and p-values. Statistical analysis was performed using the SAS 8.02 statistical software package (SAS, Heidelberg, Germany).

## Results

A total of 167 persons fulfilled the criteria for gallstone disease, corresponding to an overall prevalence of 7.8% (167/2129) (Table 1). Of the 2,129 subjects, 232 (10.9%) reported regular intake of vitamin C as powder, tablets or capsules. In this group, the prevalence of gallstones was 4.7% (n = 11). Conversely, in those not taking vitamin C supplements (n = 1897) prevalence of gallstones was 8.2% (n = 156) (Table 2).

**Table 1:** Demographic and laboratory characteristics of the study population (n = 2129)

Variable	mean (SD)	median (5^th ^- 95^th ^percentile)
**Age (years)**, n = 2129	**42.5 **(± 12.9)	**42.0 **(20 - 63)
**Height (cm)**, n = 2128	**169.4 **(± 9.3)	**169.2 **(154.5 - 185.5)
**Weight (kg)**, n = 2121	**76.2 **(± 16.0)	**74.8 **(53.9 - 104.5)
**BMI (kg/m^2^)**, n = 2120	**25.8 **(± 4.9)	**25.1 **(19.4 - 34.8)
**Waist (cm)**, n = 2120	**87.7 **(± 14.1)	**87.0 **(67.0 - 112.0)
**Hip (cm)**, n = 2120	**103.3 **(± 9.7)	**102.0 **(90.0 - 120.0)
**Waist-to-hip ratio**, n = 2120	**0.85 **(± 0.1)	**0.84 **(0.71 - 0.99)
**Total cholesterol (mmol/l)**, n = 2104	**5.5 **(± 1.1)	**5.5 **(3.9 - 7.3)
**HDL cholesterol (mmol/l)**, n = 2051	**1.6 **(± 0.5)	**1.5 **(1.0 - 2.4)
**LDL cholesterol (mmol/l)**, n = 1773	**3.3 **(± 0.9)	**3.2 **(1.8 - 5.0)
**Triglycerides (mmol/l)**, n = 1934	**1.7 **(± 1.5)	**1.3 **(0.5 - 4.4)

**Table 2:** Prevalence of gallstones (%) (number of subjects with gallstones/total number of subjects in the specific group) (n = 2129)

Age*	
18 - 30 years	1.5% (6/409)
>30 - 40 years	3.9% (12/558)
>40 - 50 years	7.8% (38/488)
>50 - 65 years	15.0% (101/674)
**Gender**	
female	10.7% (118/1104)
male	4.8% (49/1025)
**Positive family history**	
yes	12.4% (53/428)
no	6.2% (100/1609)
BMI*	
< 18.5	4.0% (2/50)
18.5 - < 25	4.0% (40/1003)
25 - < 30	8.2% (57/694)
30 - < 35	15.3% (42/275)
35 - < 40	24.7% (18/73)
≥ 40	28.0% (7/25)
**Vitamin C supplementation**	
yes	4.7% (11/232)
no	8.2% (156/1897)
**Physical activity**	
≤ 2 h/week	9.4% (111/1179)
> 2 h/week	6.0% (56/936)
**Alcohol consumption**	
yes	6.4% (95/1493)
no	11.4% (67/589)
**Caffeine consumption**	
yes	7.8% (152/1955)
no	8.1% (14/173)
Total cholesterol*	
< 4 mmol/l 4 - < 5 mmol/l	6.6% (8/122) 7.5% (41/545)
5 - < 6 mmol/l	7.7% (58/751)
≥ 6 mmol/l	8.0% (55/686)
HDL cholesterol*	
< 1 mmol/l	10.9% (32/295)
≥ 1 mmol/l	7.1% (125/1756)
LDL cholesterol*	
≥ 4 mmol/l	6.3% (21/333)
< 4 mmol/l	8.2% (118/1440)
Triglycerides*	
≥ 2.3 mmol/l	7.2% (28/390)
< 2.3 mmol/l	7.8% (120/1544)
**Vegetarian diet**	
yes	6.1% (4/66)
no	7.9% (162/2053)
**Diabetes mellitus**	
yes	23.7% (14/59)
no	7.4% (153/2064)
Waist circumference* (in cm)	
≤ 90 (f)/≤ 100 (m)	4.8% (77/1595)
> 90 (f)/> 100 (m)	17.0% (89/525)
Waist to hip ratio*	
≤ 0.8 (f)/≤ 0.9 (m)	4.2% (47/1132)
> 0.8 (f)/> 0.9 (m)	12.0% (119/988)
**Smoking**	
yes	7.0% (81/1154)
no	8.7% (85/973)

*These variables were evaluated as continuous variables in the multiple logistic regression *f (in females); m (in males)*

**Figure 1:**
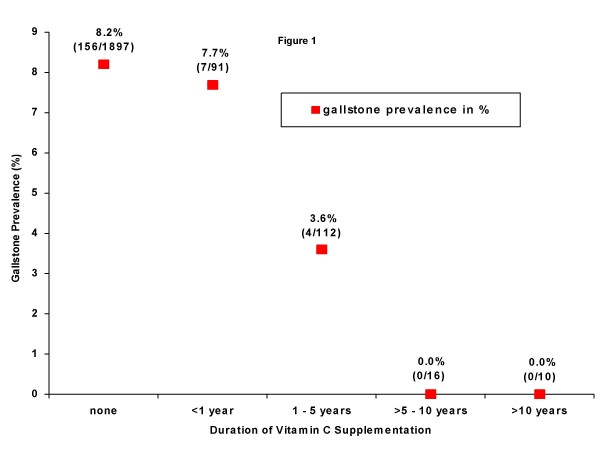
**Gallstone prevalence and duration of vitamin C supplementation (n = 2,126)**.

Study subjects with regular vitamin C supplementation were further subdivided into four predefined subgroups based on the duration of supplementation (n = 3 missing values). The longer the period of vitamin C supplementation, the lower was the proportion of subjects with gallstones (Figure 1). Subjects without (n = 1,897) and with vitamin C supplementation (n = 232) showed no remarkable differences in age, BMI, gender, family history, vegetarian diet, alcohol, tobacco, and caffeine consumption, diabetes mellitus, waist-to-hip ratio, cholesterol and triglyceride levels (Table 3).

**Table 3:** Characterization of subjects in relation to vitamin C supplementation (n = 2129)

	No Vitamin C supplementation (n = 1897)	Vitamin C supplementation (n = 232)
Age (years)*	42.4 (± 12.9)	43.4 (± 12.8)
BMI (kg/m^2^)*	25.8 (± 4.9)	25.2 (± 4.8)
Gender		
female	985 (51.9%)	119 (51.3%)
male	912 (48.1%)	113 (48.7%)
Positive family history		
yes	381 (21.0%)	47 (20.8%)
no	1430 (79.0%)	179 (79.2%)
Physical activity		
yes	811 (43.0%)	125 (54.8%)
no	1076 (57.0%)	103 (45.2%)
Vegetarian		
yes	57 (3.0%)	9 (3.9%)
no	1830 (97.0%)	223 (96.1%)
Alcohol consumption		
yes	1316 (71,1%)	177 (76.6%)
no	535 (28.9%)	54 (23.4%)
Smoking		
yes	1026 (54.1%)	128 (55.2%)
no	869 (45.9%)	104 (44.8%)
Caffeine consumption		
yes	1740 (91.8%)	215 (92.7%)
no	156 (8.2%)	17 (7.3%)
Diabetes mellitus		
yes	54 (2.9%)	5 (2.2%)
no	1838 (97.2%)	226 (97.8%)
Waist circumference (cm)*	87.8 (± 14.1)	87.0 (± 14.0)
Waist-to-hip ratio*	0.85 (± 0.09)	0.84 (± 0.09)
Total cholesterol (mmol/l)*	5.51 (± 1.07)	5.53 (± 1.04)
HDL cholesterol (mmol/l)*	1.58 (± 0.45)	1.60 (± 0.41)
LDL cholesterol (mmol/l)*	3.25 (± 0.94)	3.29 (± 0.95)
Triglycerides (mmol/l)*	1.70 (± 1.54)	1.61 (± 2.72)

*Mean (SD)

### Multiple logistic regression analyses

Multiple logistic regression analysis showed a strong association of gallstone prevalence with age (OR 1.06 per year; 95% CI, 1.04 to 1.08; P < 0.001), female gender (OR 2.78; 95% CI, 1.89 to 4.07; P < 0.001), BMI (OR 1.12 per kg/m^2^; 95% CI, 1.08 to 1.15; P < 0.001), and a family history of gallstone disease (OR 1.84; 95% CI, 1.26 to 2.69; P = 0.001) (Table 4).

**Table 4:** Multiple logistic regression of established and potential risk factors for gallstone prevalence

Variable	Odds Ratio	95% CI	P - value
**Model including established risk factors only:**			
Age (per year)	1.06	1.04 - 1.08	< 0.001
Female gender	2.78	1.89 - 4.07	< 0.001
Positive family history	1.84	1.26 - 2.69	0.001
BMI (per kg/m^2^)	1.12	1.08 - 1.15	< 0.001

**Separate models for each of the factors adjusted for the established risk factors:**			
**Vitamin C supplementation (yes)**	**0.36**	**0.16 - 0.80**	**0.01**
Vegetarian (yes)	1.19	0.41 - 3.50	0.75
Caffeine consumption (yes)	0.74	0.40 - 1.38	0.35
**Alcohol consumption (yes)**	**0.64**	**0.43 - 0.94**	**0.02**
Smoking (yes)	1.06	0.74 - 1.52	0.77
Diabetes mellitus (yes)	1.58	0.76 - 3.30	0.22
**Waist circumference (per cm)**	**1.03**	**1.01 - 1.06**	**0.03**
**Waist-to-hip ratio (per unit)**	**21.36**	**1.41 - 322.54**	**0.02**
**Total cholesterol (per mmol/l)**	**0.67**	**0.55 - 0.81**	**< 0.001**
Triglycerides (per mmol/l)	0.91	0.79 - 1.04	0.18
**HDL cholesterol (per mmol/l)**	**0.63**	**0.39 - 1.03**	**0.06**
LDL cholesterol (per mmol/l)	0.69	0.55 - 0.86	0.001*
**Leisure time physical activity** **(> 2 hours/week)**	**0.62**	**0.43 - 0.91**	**0.01**

**bold **= pre-selected risk factors with P < 0.10

* = factor not pre-selected due to large number of missing values

The influence of other potential factors was assessed after adjustment for established risk factors (age, gender, BMI and family history). These factors included regular vitamin C supplementation, smoking, alcohol and caffeine consumption, vegetarian diet, lipid concentrations, leisure time physical activity, medication, and diabetes mellitus (Table 4). Separate multiple regression analyses showed a reduced prevalence of gallstones for each of the following factors: regular vitamin C supplementation (OR 0.36; 95% CI, 0.16 to 0.80; P = 0.01), alcohol consumption (OR 0.64; 95% CI, 0.43 to 0.94; P = 0.02), leisure time physical activity (OR 0.62; 95% CI, 0.43 to 0.91; P = 0.01), elevated total cholesterol (OR 0.67 per mmol/l; 95% CI: 0.55 to 0.81; P < 0.001), LDL cholesterol (OR 0.69 per mmol/l; 95% CI, 0.55 to 0.86; P = 0.001), and HDL cholesterol levels (OR 0.63 per mmol/l; 95% CI, 0.39 to 1.03; P = 0.07).

Factors associated with increased gallstone prevalence included waist circumference (OR 1.03; 95% CI, 1.01 to 1.06; P = 0.03), and waist-to-hip ratio (OR 21.36; 95% CI, 1.41 to 322.54; P = 0.02). After adjustment for established risk factors, all other factors such as diabetes mellitus, vegetarian diet, caffeine consumption, smoking, and triglycerides were not associated with the prevalence of gallstones (Table 4).

### Multiple logistic regression analysis with backward elimination

Potential risk factors with P values < 0.1 were pre-selected and included in a multiple logistic regression with backward elimination, whereby age, gender, BMI, and family history were forced into the model (Table 5).

**Table 5:** Results of multiple logistic regression analysis with backward elimination of potential factors after adjustment for established risk factors

Variable	Odds Ratio	95% CI	P - value
Factors forced into the model:			
Age (per year)	1.07	1.05 - 1.10	< 0.001
Female gender (yes)	3.74	2.15 - 6.51	< 0.001
Positive family history (yes)	1.82	1.21 - 2.76	0.005
BMI (per kg/m^2^)	1.04	0.95 - 1.14	0.37

Selected risk factors:			
Vitamin C supplementation (yes)	0.34	0.14 - 0.81	0.01
Physical activity (> 2 hours/week)	0.62	0.42 - 0.94	0.02
Cholesterol (per mmol/l)	0.65	0.52 - 0.79	< 0.001

In this model, waist-to-hip ratio (P = 0.88), HDL cholesterol (P = 0.80), waist circumference (P = 0.23), and alcohol consumption (P = 0.21) were eliminated through backward elimination. Only regular vitamin C supplementation (OR 0.34; 95% CI, 0.14 to 0.81; P = 0.01), physical activity (OR 0.62; 95% CI, 0.42 to 0.94; P = 0.02) and total cholesterol (OR 0.65 per mmol/l; 95% CI, 0.52 to 0.79; P < 0.001) remained in the model (Table 5).

## Discussion

To our knowledge, this is the first survey in a randomly selected population sample to investigate the relationship between vitamin C supplement use and gallstone prevalence. Regarding protective factors, we found the strongest association with lower gallstone prevalence for subjects with regular vitamin C supplementation. As in other surveys, data from our study confirmed established risk factors for gallstone disease such as female gender, age, BMI, and a positive family history [23-25]

To date, observational studies partly conducted in selected populations, and studies relying on self-reported gallstone disease have suggested an association between vitamin C intake and gallstone prevalence [14-16,26]. In those studies, where the diagnosis of cholelithiasis was based solely on patients' reported history with no corroborating imaging data, missing data on asymptomatic gallstones might have resulted in a bias.

A small case-control study showed an association between dietary vitamin C intake and gallstone disease in women, which, however, was no longer statistically important in multiple logistic regression analysis [14]. Simon et al. reported an association between vitamin C supplementation and the prevalence of gallbladder disease and cholecystectomy in women with coronary heart disease [26].

In a subanalysis of NHANES II, a U-shaped relationship between subjects' ascorbic acid levels and the prevalence of reported gallbladder disease in women was shown [15]. Data from NHANES III showed an inverse relationship between ascorbic acid levels and gallbladder disease in women [16].

In addition to vitamin C intake, regular physical activity might play a role in gallstone disease. Leitzmann et al. have shown that regular physical activity reduces the risk of cholecystectomy in women [27]. Other studies have shown a reduced gallbladder stone prevalence in physically active subjects [28-31]. In a prospective study, in which 45,813 male health professionals participated, those engaging in an average of two to three hours of moderate running per week seemed to reduce their risk for newly symptomatic gallstone disease by 20-40%. [28]. Similarly, our study shows a clear inverse association between physical activity and the prevalence of gallstones, with a 40% reduced risk in those being active more than two hours per week (OR 0.62; 95% CI, 0.42 to 0.94; P = 0.02).

Our data also showed a reduced prevalence of gallstones for higher total cholesterol concentrations. However this effect, was less pronounced than for vitamin C supplementation. Descriptive analysis revealed an increase in the prevalence of gallstones with increasing cholesterol concentrations. Multiple logistic regression, however, showed the opposite direction after adjusting for age, gender, BMI, and family history of gallstone disease. As in our study, this inverse association between plasma cholesterol concentration and gallstone prevalence was shown in other studies, although the data on the relationship of dietary cholesterol and cholesterol gallstones is not conclusive [32-34].

### Limitations of the study

A limitation of the present study that has to be considered is the lack of plasma ascorbic acid level measurements. Whether a single determination of plasma levels of vitamin C, however, reflects regular vitamin C intake is controversial. A meta-analysis has shown that different groups of subjects require different amounts of vitamin C in order to achieve the same plasma level [35,36]. The determination and comparison of plasma vitamin C levels measured in different laboratories also remains problematic [37].

Since multiple determinations of plasma vitamin C levels for assessing subjects' actual vitamin C status were not possible in our study, we opted against obtaining an one-time determination. It can be assumed that subjects with regular intake of vitamin C preparations would show adequate plasma concentrations [35]. In subjects without vitamin C supplementation, plasma levels depend directly on their dietary vitamin C intake. Since this can be quite variable, there is no reliable way to predict plasma levels of vitamin C. As it is difficult to assess the intake of natural vitamin C in a large population sample, no detailed information on vitamin C rich diets apart from the precisely documented data on supplementation was recorded in our study.

Considering the possibility that, in subjects without regular vitamin C supplementation, there might exist a subgroup taking a natural diet rich in vitamin C, the difference in gallstone prevalence between subjects with high vitamin C intake (natural or through supplementation) and subjects with low vitamin C intake might be even more pronounced.

## Conclusion

Our study provides evidence that regular vitamin C supplementation is associated with reduced prevalence of gallstones. This observation is supported by data from animal experiments reported in the literature. A biochemical relationship has also been identified. Thus, it seems reasonable to postulate a possible protective effect of vitamin C on the formation of gallstones.

## Competing interests

The authors declare that they have no competing interests.

## Authors' contributions

Conception and design: TW, MMH, MK, PK, IP, BOB, WKo, WKr. Collection and assembly of data: TW, MMH, GS, IP, WKo, WKr. Analysis and interpretation of data: TW, MMH, MK, BH, RAM, DW, GA, BOB, WKo, WKr. Drafting of the article: TW, MMH, MK, RAM, DW, BOB, WKo, WKr. Critical revision of the article for important intellectual content: TW, MMH, MK, BH, RAM, DW, GS, PK, IP, GA, BOB, WKo, WKr. Statistical expertise: TW, MMH, MK, BH, WKo, WKr. Obtaining of funding: MMH, PK, IP, WKo, WKr. Administrative, technical, or logistic support: RAM, GS, PK, BOB, WKr.

All authors read and approved the final manuscript.

## Pre-publication history

The pre-publication history for this paper can be accessed here:

http://www.biomedcentral.com/1471-230X/9/74/prepub
